# Eye movement disorders and neurological symptoms in late‐onset inborn errors of metabolism

**DOI:** 10.1002/mds.27484

**Published:** 2018-11-28

**Authors:** Lisette H. Koens, Marina A.J. Tijssen, Fiete Lange, Bruce H.R. Wolffenbuttel, Alessandra Rufa, David S. Zee, Tom J. de Koning

**Affiliations:** ^1^ University of Groningen, University Medical Center Groningen, Department of Neurology Groningen The Netherlands; ^2^ University of Groningen, University Medical Center Groningen, Department of Clinical Neurophysiology Groningen The Netherlands; ^3^ Department of Endocrinology University of Groningen, University Medical Center Groningen Groningen The Netherlands; ^4^ Department of Medicine Surgery and Neurosciences University of Siena, Eye tracking and Visual Application Lab (EVA Lab)–Neurology and Neurometabolic Unit Siena Italy; ^5^ Department of Neuroscience, Department of Ophthalmology The Johns Hopkins University, The Johns Hopkins Hospital, Department of Neurology, Department of Otolaryngology‐Head and Neck Surgery Baltimore Maryland USA; ^6^ University of Groningen, Division of Metabolic Diseases, University Medical Center Groningen Groningen The Netherlands; ^7^ University of Groningen, Department of Genetics, University Medical Center Groningen Groningen The Netherlands

**Keywords:** eye movement disorders, inborn errors of metabolism, movement disorders, adult‐onset

## Abstract

Inborn errors of metabolism in adults are still largely unexplored. Despite the fact that adult‐onset phenotypes have been known for many years, little attention is given to these disorders in neurological practice. The adult‐onset presentation differs from childhood‐onset phenotypes, often leading to considerable diagnostic delay. The identification of these patients at the earliest stage of disease is important, given that early treatment may prevent or lessen further brain damage. Neurological and psychiatric symptoms occur more frequently in adult forms. Abnormalities of eye movements are also common and can be the presenting sign. Eye movement disorders can be classified as central or peripheral. Central forms are frequently observed in lysosomal storage disorders, whereas peripheral forms are a key feature of mitochondrial disease. Furthermore, oculogyric crisis is an important feature in disorders affecting dopamine syntheses or transport. Ocular motor disorders are often not reported by the patient, and abnormalities can be easily overlooked in a general examination. In adults with unexplained psychiatric and neurological symptoms, a special focus on examination of eye movements can serve as a relatively simple clinical tool to detect a metabolic disorder. Eye movements can be easily quantified and analyzed with video‐oculography, making them a valuable biomarker for following the natural course of disease or the response to therapies. Here, we review, for the first time, eye movement disorders that can occur in inborn errors of metabolism, with a focus on late‐onset forms. We provide a step‐by‐step overview that will help clinicians to examine and interpret eye movement disorders. © 2018 The Authors. *Movement Disorders* published by Wiley Periodicals, Inc. on behalf of International Parkinson and Movement Disorder Society.

Inborn errors of metabolism (IEM) are a heterogeneous group of genetic disorders that cause dysfunction of an enzyme or transporter involved in cellular metabolism. Historically, inborn errors were thought to be rare, occuring in less than 1 per 100,000 live births and to only present during infancy or early childhood.^1^ We now know that this prevalence is an underestimate, and that IEM present in adolescence or adulthood much more often than previously thought. Retrospective data from an ethnically diverse population in the United Kingdom (1999‐2003) revealed an overall prevalence of metabolic disease of 1 per 784 live births; mitochondrial diseases, lysosomal storage diseases, and amino acid disorders were most frequent. Furthermore, more than one quarter of all diagnoses were made after the age of 15 years.^2^ Adult phenotypes may differ from the classic childhood‐onset phenotypes. In adulthood, many IEM patients present with neurological or psychiatric symptoms, but considering an IEM in the differential diagnosis of an adult patient is still uncommon among neurologists.^3^ Missing or delaying diagnosis of an IEM can have important implications. In particular, patients with a milder phenotype appear to benefit most from timely treatment, so identifying them is important to prevent further (neurological) damage.^4^

Whereas the neurological symptoms in patients with IEM often involve various types of movement disorders,^5^ eye movement disorders are also frequently observed and can be an important diagnostic clue.^6^ The type of eye movement disorder can often further delineate the type of IEM.

The aim of this article is to review the abnormalities of eye movements that can be observed in IEM, with an emphasis on those IEMs that can present later in life. Our goal is to increase awareness of eye movements in adult patients with movement disorders and other neurological or psychiatric disturbances because they can be the key to early diagnosis. Because more types of IEM are being identified that can be treated, early recognition of these disorders is important.

## Search Strategy and Selection Criteria

We reviewed articles regarding ocular motility disorders and IEM up to June 2017. References were identified by PubMed, text book search, and through citations in relevant articles and books. Only articles published in English were included. Search terms are in Supplementary Appendix I. Only IEMs with at least 2 patients with some type of eye movement disorder were included in the review. Although we focused on late‐onset IEM (adolescent‐onset 16‐18 years of age, adult‐onset > 18 years of age), it is difficult to discriminate specifically between early‐ and late‐onset forms, given that eye movement disorders are frequently not described in the literature. For that reason, children were also included in this review. We included mitochondrial diseases as a combined disease group instead of specific subtypes. We excluded articles in which nystagmus secondary to blindness was the only ocular motor finding. Supplementary Appendix II presents a list of references with videos of eye movement disorders in IEM.

## Examination of Eye Movements

Disorders of eye movement can be categorized as peripheral and central (Table 1). Peripheral eye movement disorders are particularly frequent in mitochrondrial disorders and commonly affect the two eyes differently, resulting in ocular misalignment and diplopia. Progressive external ophthalmoplegia (PEO) is an important exception to this rule.^7^ Because of the insidious onset and slow progression, patients do not complain of diplopia and may be unaware of any disorder of eye movements.^8^ Central causes of ocular motor abnormalities usually affect both eyes.^7^, ^9^

**Table 1:** Peripheral and central eye movement disorders

	Anatomical Location	Abnormality During Examination
Peripheral origin	Retina and optic nerve	Nystagmus
	Ocular muscles, ocular nerves and nuclei (oculomotor, trochlear, abducens)	Impaired range of motion, strabismus, abnormal smooth pursuit, saccades, vestibulo‐ocular reflex, and optokinetic nystagmus
	Peripheral vestibular system	Nystagmus
Central origin	Central visual pathways and cortex	Nystagmus
	Cerebellum and brainstem	Impaired range of motion, abnormal smooth pursuit, saccades, vestibulo‐ocular reflex, and optokinetic nystagmus
	Frontoparietal cortex, basal ganglia, and cerebellum	Oculomotor apraxia^a^, impaired smooth pursuit
	Basal ganglia and midbrain	Oculogyric crisis^b^

^a^ Oculomotor apraxia: failure of saccade initiation.

^b^ Oculogyric crisis: episodes of tonic upward conjugate deviation of the eyes with an inability to look downward.

Clinical examination of the eye movements in all patients with a suspected IEM is essential. Patients themselves may not report visual symptoms or may only have nonspecific complaints.^9^, ^10^ Table 2 provides a short step‐by‐step overview of the clinical examination of eye movements. Every step in the table needs to be followed because different types of eye movement disorders can exist in 1 patient.

**Table 2:** Seven‐step examination of eye movements (modified from both Leigh and Strupp^9^, ^10^)

Type of Examination		Abnormalities
Step 1		
Inspection	Posture of head and neck	Head turn or tilt in diplopia, gaze palsy, dystonia, or otolith disorders. Head thrusts in saccade initiation disorders (ocular motor apraxia)
	Face and eyelids	Blepharospasm, ptosis
Step 2		
Examination of vision, pupils, and fundus	Vision	Impaired visual acuity
	Confrontation visual fields	Visual field defect
	Pupillary reflexes	Delayed or absent, light to near dissociation
	Fundus	Pathology of the retina or optic nerve, subtle forms of nystagmus or saccadic oscillations
Step 3		
Fixation, range of motility, and alignment	Gaze in center and eccentric position, Vergence	Limited range of motion of one or both eyes Strabismus (horizontal and vertical misalignment) Saccadic intrusions (square‐wave jerks) during fixation Gaze palsy Primary position or gaze‐evoked nystagmus Oculogyric crises
Step 4		
Frenzel's spectacles to eliminate fixation or occlusive ophthalmoscopy (cover one eye while viewing the fundus of the other)	Gaze in center and eccentric position	Spontaneous nystagmus brought out by removal of fixation
Step 5		
Saccades	Rapidly changing gaze from one fixation point to another ‐Spontaneous saccades ‐Self‐generated saccades on ‐command (look left‐right, up‐down) ‐Saccades to specific targets (pen‐finger) ‐Rapid self‐paced saccades between two specific targets ‐Antisaccades (look at the mirror location in the opposite direction to the target when it appears or moves)	Impaired initiation: ocular motor apraxia Increased latency, decreased velocity, inaccuracy, or disconjugacy
Step 6		
Optokinetic nystagmus and pursuit	Tracking a small, smoothly moving target in horizontal and vertical directions with head still or with head moving (vestibular suppression)	Corrective, catch‐up saccades (tracking with head still) or back‐up saccades (tracking with head moving) to reacquire the fixation target
	Optokinetic drum or tape in horizontal and vertical direction	Not inducible, direction asymmetry
Step 7		
Vestibulo‐ocular reflex	Head‐impulse test^a^ (passive rapid turning of the head while fixation on a stationary target)	Corrective saccades instead of stable fixation
	Head‐shaking test (oscillate head 20 times)	Post head shaking nystagmus in peripheral and in central vestibular disorders

^a^ The head impulse test maximizes the ability of the vestibular system to overcome a supranuclear palsy.

Video‐oculography (VOG) allows for better quantitative documentation of abnormalities. Typically, a digital camera mounted in goggles uses the contrast between the pupil and iris to track the movement and position of one or both of the eyes.^11^ VOG is an effective user‐ and patient‐friendly tool to quantify eye movements, including subtle changes in latency, velocity, and accurary of saccades.^12^ It can be used to support or make a diagnosis and measure effectiveness of treatment during follow‐up. For example, in Niemann‐Pick type C (NP‐C), saccadic parameters measured by VOG have been reported to be a robust indicator of efficacy of treatment with Miglustat.^13^

## Inborn Errors of Metabolism Associated With Ocular Motor Disorders

We will discuss abnormalities of eye movements in IEM. An overview of the various IEMs associated with ocular motor disorders, including the underlying gene defect, metabolic abnormalities, age of onset, early‐onset symptoms, and treatment, is given in Table 3. Table 4 presents the described eye movement disorders for each of those IEMs.

**Table 3:** Inborn errors of metabolism associated with eye movement abnormalities

	Gene	Inheritance	Functional Consequences	Age of Onset	Early‐Onset Symptoms	Treatment
**Lysosomal storage diseases**						
Niemann‐Pick type C	NP‐C1, NP‐C2	AR	Lipid accumulation in cells	Early infantile‐adulthood	Hepatosplenomegaly, neuropsychiatric symptoms later in life	Miglustat
Gaucher's disease	GBA	AR	Accumulation of glucosylceramide and the cytotoxic derivative of glucosylceramide	Early infantile‐adulthood	Type 1: hepatosplenomegaly, bone anomalies, cytopenia Type 2: early death attributed to neurological symptoms Type 3: progressive encephalopathy and systemic symptoms	Miglustat, enzyme replacement therapy
Tay‐Sachs disease	HEXA	AR	Disturbance of catabolism and eventually accumulation of GM2 ganglioside, particularly in neurons	Usually infantile, sometimes late‐onset	Early death attributed to psychomotor retardation, neurodegeneration, and muscle weakness Cherry‐red spot in the ocular fundus	
Sandhoff's disease	HEXB	AR	Disturbance of catabolism and eventually accumulation of GM2 ganglioside, particularly in neurons	Usually infantile, sometimes late‐onset	Early death attributed to psychomotor retardation, neurodegeneration, and muscle weakness Cherry‐red spot in the ocular fundus	
**Disorders of lipid metabolism**						
Abetalipoproteinemia	MTTP	AR	Impairment of the absorption of dietary fats, cholesterol, and fat‐soluble vitamins	Childhood, sometimes late‐onset	Failure to thrive and growth failure, hepatomegaly with steatosis, diarrhea, ataxia	High‐dose vitamin E, supplementation of vitamin A,D, and K, low‐fat diet
Cerebrotendinous xanthomatosis	CYP27A1	AR	Cholesterol and cholestanol accumulation in cells	Childhood‐adulthood	Diarrhea, jaundice, premature cataracts, ataxia; xanthomata in the second or third decade	Chenodeoxycholic acid and statin therapy
**Disorders of carbohydrate metabolism**						
Glucose transporter type 1 deficiency	SLC2A1	AD	Impairment of the transport of glucose from the blood to cerebral tissue	Infantile‐childhood, sometimes late‐onset	Psychomotor retardation, seizures, microcephaly, spasticity, movement disorders	Ketogenic diet
**Disorders of mineral, metal, and vitamin metabolism**						
Wilson's disease	ATP7B	AR	Accumulation of copper, particularly in the liver and brain	Early childhood‐adulthood	Liver disease, neurological and psychiatric symptoms, Kayser‐Fleischer ring	Penicillamine, trientine, zinc
Hypermanganesemia with dystonia 1	SLC30A10	AR	Accumulation of manganese in liver and basal ganglia	Childhood‐adolescence	Severe dystonia and other movement disorders, liver dysfunction	Chelation therapy, iron supplementation
Pantothenate kinase‐associated neurodegeneration	PKAN2	AR	Accumulation of iron, especially in the globus pallidus	Childhood‐adolescence	Dystonia, chorea, rigidity, dysarthria, pigmentary retinopathy, developmental delay	
Adult‐onset dystonia‐parkinsonism	PLA2G6	AR	Accumulation of iron, especially in the globus pallidus	Adulthood	Parkinsonism, dystonia, cognitive decline	
Biotin‐thiamine–responsive basal ganglia disease	SLC19A3	AR	Impairment of thiamine uptake into cells, causing destruction of the head of the caudate and putamen	Infantile‐adolescence	Acute dystonia, encephalopathy	Thiamine and biotin
Ataxia with vitamin E deficiency	TTPA	AR	Impairment of incorporation of vitamin E into very‐low‐density lipoprotein, leading to low plasma levels of vitamin E	Childhood‐adulthood	Ataxia, areflexia, impaired proprioception	Vitamin E
**Disorders of amino acid metabolism**						
Maple syrup urine disease	BCKDHA, BCKDHB, DBT	AR	Preventing the normal breakdown of leucine, isoleucine, and valine, leading to accumulation of these amino acids	Infantile, late‐onset presentation is rare	Maple syrup odor in the cerumen and later in urine, poor feeding, progressive encephalopathy, intermittent apnea, stereotyped movements	Low‐protein diet, leucine‐, isoleucine‐, and valine‐free amino acid supplement, emergency regimens
Glutaric aciduria type 1	GCDH	AR	Preventing the breakdown of lysine, hydroxylysine, and tryptophan, leading to accumulation of metabolites	Childhood‐adulthood	Acute encephalopathic crises, dystonia (with insidious onset)	Avoidance and treatment of triggers, dietary lysine restriction, L‐carnitine, emergency regimens
**Congenital disorders of glycosylation**						
Phosphomannomutase 2 deficiency	PMM2	AR	Affecting glycoprotein biosynthesis	Childhood, sometimes late‐onset	Developmental delay, hypotonia, ataxia, retinitis pigmentosa, strabismus, seizures, abnormal fat distribution	
**Disorders of purine or pyrimidine metabolism**						
Lesch‐Nyhan syndrome	HPRT	X‐linked recessive	Affecting the breakdown of purines, leading to high levels of uric acid in blood	Infantile‐adulthood	Severe dystonia and behavioral abnormalities, including self‐injury	Treatment of hyperuricemia
**Peroxisomal disorders**						
Zellweger spectrum disorders	PEX genes	AR	Affecting the formation of functional peroxisomes	Infantile, sometimes late‐onset	Hypotonia, seizures, deafness, developmental delay, characteristic facial appearance, liver dysfunction	
**Neurotransmitter disorders**						
Aromatic L‐amino acid decarboxylase deficiency	DDC	AR	Reduced production of dopamine, serotonin, and tryptamine	Usually infantile, sometimes late‐onset	Dystonia, psychomotor retardation, spasticity, autonomic dysfunction	Pyridoxine, dopamine agonists, and MAO‐inhibitors
Tyrosine hydroxylase deficiency	TH	AR	Impairment of the conversion of L‐tyrosine to l‐dopa	Usually infantile, sometimes late‐onset	Dystonia, mild intellectual deficit	l‐dopa
GTP‐CH‐I deficiency	GCH1	AR or AD	Impairment of the tetrahydrobiopterin (BH4) biosynthesis	Infantile‐adolescence	Psychomotor retardation, convulsions, drowsiness, abnormal movements, autonomic dysfunction	l‐dopa, 5‐hydroxytryptophan, and tetrahydrobiopterin (the latter two only in recessive forms)
Sepiapterin reductase deficiency	SPR	AR (or AD?)	Impairment of the tetrahydrobiopterin (BH4) biosynthesis	Usually infantile	Dystonia, psychomotor retardation, axial hypotonia, weakness	l‐dopa, 5‐hydroxytryptophan, and tetrahydrobiopterin
6‐Pyruvoyl‐tetrahydropterin synthase deficiency	PTS	AR	Impairment of the tetrahydrobiopterin (BH4) biosynthesis	Usually infantile, sometimes late‐onset	Psychomotor retardation, convulsions, drowsiness, abnormal movements, autonomic dysfunction	l‐dopa, 5‐hydroxytryptophan, and tetrahydrobiopterin
Brain dopamine‐serotonin vesicular transport disease	SLC18A2	AR	Impairment of the monoamine transport in synaptic vesicles	Usually infantile, sometimes late‐onset	Dystonia‐parkinsonism, autonomic dysfunction, developmental delay	Dopamine agonists
Dopamine transporter deficiency syndrome	SLC6A3	AR	Loss of function of the presynaptic dopamine transporter	Usually infantile	Dystonia‐parkinsonism, developmental delay	
**Energy metabolism disorders**						
Mitochondrial diseases	Multiple genes	AR, AD, X‐linked	Impairment of the respiratory chain or oxidative phosphorylation system	Infantile‐adulthood	Wide range of symptoms, including movement disorders, psychomotor retardation or regression, epilepsy, muscle weakness, migraine	
Pyruvate dehydrogenase E2 deficiency	DLAT	AR	Accumulation of pyruvate in cells, resulting in production of lactic acid and alanine	Usually infantile, sometimes late‐onset	Severe neonatal lactic acidosis, leading to death, dystonia	Ketogenic diet, thiamine, triheptanoin

**Table 4:** Abnormalities of eye movements in inborn errors of metabolism

						Impaired Saccades							
	Ptosis	Impaired Vision	Oculogyric Crisis	Nystagmus	Saccadic Oscillations (Flutter and Opsoclonus)	*Paralysis*	*Dysmetric*	*Slow*	Ocular Motor Apraxia (Saccade Initiation Deficit)	Impaired Smooth Pursuit	Impaired Optokinetic Nystagmus	Impaired Vestibulo‐ ocular Reflex	Estimated Prevalence of Eye Movement Disorders
**Lysosomal storage diseases**													
Niemann‐Pick type C						x(V)		x		x	x		+
Gaucher's disease type 1								x					?
Gaucher's disease type 2						x		x	x	x	x		+
Gaucher's disease type 3						x(H)		x	x	x	x	x	+
Tay‐Sachs disease, infantile form		x				x(V)				x			+/–
Tay‐Sachs disease, late‐onset form						x(V)	x	x		x	x		+/–
Sandhoff's disease		x		x			x	x		x	x		?
**Disorders of lipid metabolism**													
Abetalipoproteinemia	x	x		x				x		x	x	x	+
Cerebrotendinous xanthomatosis		x					x	x		x			+/–
**Disorders of carbohydrate metabolism**													
Glucose transporter type 1 deficiency	x		x		x								+
**Disorders of mineral, metal, and vitamin metabolism**													
Wilson's disease		x	x			x(V)	x	x		x	x	x	+
Hypermanganesemia with dystonia 1							x	x					?
Pantothenate kinase‐associated neurodegeneration		x	x				x	x		x	x	x	+/–
Adult‐onset dystonia‐parkinsonism		x	x	x		x(V)		x		x			+/–
Biotin‐thiamine–responsive basal ganglia disease	x					x(V)				x			?
Ataxia with vitamin E deficiency		x		x		x		x	x	x			+
**Disorders of amino acid metabolism**													
Maple syrup urine disease	x				x	x				x		x	+/–
Glutaric aciduria type 1		x		x		x(V)				x			+/–
**Congenital disorders of glycosylation**													
Phosphomannomutase 2 deficiency		x		x	x				x	x	x	x	+
**Disorders of purine or pyrimidine metabolism**													
Lesch‐Nyhan syndrome								x	x	x			+/–
**Peroxisomal disorders**													
Zellweger spectrum disorders		x		x				x		x			+
**Neurotransmitter disorders**													
Dopamine transporter deficiency syndrome			x		x			x	x	x			+
Other disorders of dopamine synthesis or transport^a^			x										+/–
**Energy metabolism disorders**													
Mitochondrial diseases	x	x		x		x(PEO)	x	x		x	x	x	+
Pyruvate dehydrogenase E2 deficiency	x			x		x(V)			x				?

x: Present.

^a^ Aromatic L‐amino acid decarboxylase deficiency, tyrosine hydroxylase deficiency, GTP‐CH‐I deficiency (dominant and recessive), sepiapterin reductase deficiency, 6‐pyruvoyl‐tetrahydropterin synthase deficiency, brain dopamine‐serotonin vesicular transport disease.

(V): Primarily vertical. (H): Primarily horizontal. (PEO): Progressive external ophthalmoplegia.

+: frequent. + /–: infrequent. ?: unknown.

### Lysosomal Storage Diseases

Late‐onset NP‐C usually presents with neurological problems. Movement disorders are frequent, particularly in these adolescent‐ and adult‐onset forms.^14^ Eye movement disorders are also an important feature. Vertical supranuclear gaze palsy (VSGP) is a key feature and is present in approximately 65% of patients.^15^ VSGP in patients with NP‐C is characterized by a paralysis of vertical (especially downward) saccades, whereas smooth pursuit is initially spared.^16^ Horizontal saccades are initially preserved, but are ultimately affected as the disease progresses.^17^, ^18^ A so‐called round‐the‐houses phenomenon occurs when attempting vertical saccades: The eyes do not move directly up and down, but in a lateral arc (Video 1).^16^, ^19^ A similar phenomenon occurs during horizontal saccades in Gaucher's disease. No abnormalities of vestibulo‐ocular responses have been found.^20^ Treatment is possible with Miglustat.^21^

Gaucher's disease type 2 (acute neurological form) and type 3 (subacute neurological form) are the neuronopathic forms of this lysosomal storage disorder. Gaucher's disease type 2 presents during infancy and abnormalities of eye movements are early signs in affected children, including ocular motor paralysis, slowness of saccades, oculomotor “apraxia,” and strabismus.^22^, ^23^ Gaucher's disease type 3 presents during childhood or adolescence. Movement disorders are common in type 3, particularly ataxia and parkinsonism.^24^ However, patients often present with (myoclonus) epilepsy and supranuclear gaze palsy that only affects horizontal gaze.^25^, ^26^, ^27^ Horizontal saccades are markedly slow and may show a curved trajectory, whereas vertical saccades are initially preserved.^28^, ^29^, ^30^ Vestibulo‐ocular responses may be impaired.^31^ Ocular motor apraxia, which in fact reflects abnormal patterns of head motion associated with defects in initiation of saccades, is also observed in Gaucher's types 2 and 3.^30^, ^32^ Similar to NP‐C, patterns of abnormal saccades can be used to monitor progression of disease.^9^, ^33^ Gaucher's disease type 1 is the chronic non‐neurological form; however, subtle slowness of saccades has been reported in some patients.^22^, ^34^, ^35^, ^36^ Enzyme replacement therapy and substrate reduction therapy are available.^21^

In the late‐onset form of Tay‐Sachs disease (GM2 gangliosidosis), motor symptoms are frequent. These are caused by motor neuron dysfunction and cerebellar involvement with ataxia.^37^ Abnormalities of eye movements are not a classic feature of late‐onset Tays‐Sachs disease, but impaired smooth pursuit with square‐wave jerks (saccadic intrusions), transient decelerations of saccades, and up‐gaze palsy have been described. Vestibulo‐ocular responses are normal.^37^, ^38^, ^39^, ^40^ In early‐onset Tach‐Sachs disease, vertical gaze is impaired early and horizontal gaze later in the disease.^9^ Treatment is not available.

The clinical picture of Sandhoff's disease (GM2 gangliosidosis) is similar to Tay‐Sachs disease. Early childhood forms are most common. Late‐onset forms of Sandhoff's disease are rare and have a milder phenotype. They often present as a complex neurological disorder with ataxia, chorea, tremor, dystonia, or parkinsonism in combination with motor neuron dysfunction.^21^ Abnormalities of eye movements include impaired horizontal and vertical saccades with nystagmus.^41^, ^42^, ^43^ A patient with adult‐onset Sandhoff's disease and pendular nystagmus in combination with palatal tremor has been described.^41^ Treatment is not available.

### Disorders of Lipid Metabolism

Signs of *abetalipoproteinemia* occur early in life and progress with time. Neurological manifestations resulting from vitamin deficiency often begin in the first or second decade of life. Low vitamin E in particular can cause progressive neurological symptoms affecting the peripheral and central nervous system. Adult patients show malabsorption, steatosis, abnormal liver transaminases, and neurological signs.^21^ Abnormalities of eye movements are typical, including progressive gaze disturbances attributed to paresis of the medial rectus muscles and a characteristic pattern of dissociated nystagmus. The latter consists of an intense nystagmus, but with limited range in the adducting eye, and a less‐intense nystagmus, but with full range, in the abducting eye. Patients complain of trouble reading and of difficulties associated with impaired convergence. Saccades are slow and hypometric. Vestibular nystagmus and optokinetic nystagmus have abnormal or absent quick phases.^44^ A low‐fat diet with reduced long‐chain fatty acids and fat‐soluble vitamin supplements is recommended.^21^

Late‐onset cerebrotendinous xanthomatosis is characterized by tendon xanthomas, psychiatric symptoms, and neurological symptoms, including pyramidal, cerebellar, and extrapyramidal signs in the second or third decade of life.^21^, ^45^ Patients show abnormal pursuit, increased saccadic intrusions, multistep saccades, and antisaccade deficits.^46^ Chenodeoxycolic acid and statin therapy are an effective treatment and can prevent neurological involvement if started early.^21^, ^47^, ^48^

### Disorders of Carbohydrate Metabolism

Symptoms of glucose transporter type 1 deficiency usually occur early in life, but may present in adolescence or adulthood. In the late presentation form, paroxysmal exercise‐induced dyskinesia occurs that predominantly manifests as dystonia, chorea, and ballism. Epilepsy is also observed.^49^ Abnormalities of eye movements are common and may be highly characteristic brief multidirectional paroxysmal episodes of rapid eye movements in combination with head movements in the same direction, a phenomenon called aberant gaze saccades.^50^ Eye rolling and fluttering, strabismus, opsoclonus, and limitation of vertical eye movements have also been described.^50^, ^51^, ^52^ Early diagnosis is important because this disorder can be treated with a ketogenic diet.^21^

### Disorders of Mineral, Metal, or Vitamin Metabolism

Symptoms of Wilson's disease often begin in the teenage years. Liver disease is frequently the presenting sign, but psychiatric and neurological symptoms including movement disorders are also frequent presentations. The Kayser‐Fleischer ring, copper deposits that form a ring in the cornea, is the ophthalmological hallmark of Wilson's disease.^21^ Abnormalities of eye movements are frequently present. Impaired vertical, but sometimes also horizontal pursuit, selective slowing of downward saccades, and dysmetria of saccades are all reported.^53^, ^54^, ^55^, ^56^, ^57^ Gaze distractibility has also been described in which patients cannot fix their eyes on a stationary or moving object for more than a few seconds without being distracted by other stimuli.^58^ At least 1 patient with oculogyric crises has been reported on.^59^ Treatment is possible with chelation therapy.^21^

Adult‐onset hypermanganesemia with dystonia 1 is characterized by parkinsonism, whereas children usually present with dystonia. Bilateral hyperintensities in the basal ganglia and white matter attrobuted to accumulation of manganese are typically observed on brain imaging.^21^ Increased latency of saccades, misdirected antisaccades, and multistep saccades have been observed by one of the authors (A.R., personal observations). Chelation therapy and iron supplementation are recommended^21^.

Pantothenate kinase‐associated neurodegeneration (or NBIA type 1) is the most common form of neurodegeneration with brain iron accumulation (NBIA). This is reflected in the “eye‐of‐the‐tiger” sign on brain MRI.^60^ Late‐onset disease occurs during the second or third decade. It is slowly progressive and is characterized by speech problems, movement disorders, and psychiatric symptoms.^61^ Horizontal and vertical supranuclear gaze palsy, impaired saccades, abnormal optokinetic nystagmus, and impaired horizontal vestibulo‐ocular responses have been described.^62^, ^63^ Oculogyric crisis has been reported in 1 patient.^60^ Treatment with chelation therapy is not effective.^21^

Adult‐onset dystonia‐parkinsonism (NBIA type 2) also belongs to the heterogeneous group of degenerative disorders causing iron accumulation. Adults usually present before the age of 30 and have parkinsonism, dystonia and cognitive decline. Ophthalmic features include strabismus, up‐gaze palsy, impaired pursuit with saccadic intrusions, and pendular nystagmus. Vestibulo‐ocular responses are not impaired.^64^ A case of oculogyric crisis induced by levodopa has been described in a patient with adult‐onset dystonia‐parkinsonism.^65^ Only symptomatic treatment is available.

The onset of biotin‐thiamine‐responsive basal ganglia disease is usually during early childhood, but can occur later in life.^21^, ^66^ In addition to acute dystonia and encephalopathy, bilateral external ophthalmoplegia is observed.^67^, ^68^, ^69^ Diagnosis is important because treatment with thiamine and biotin can be life‐saving.

Finally, onset of ataxia with vitamin E deficiency can be at any age. Symptoms include ataxia, areflexia, and impaired proprioception. Nystagmus is observed as part of a cerebellar syndrome. Impaired smooth pursuit, slow saccades, ocular motor apraxia, and strabismus have been reported.^9^, ^70^, ^71^, ^72^ Treatment is with high‐dose vitamin E supplementation.^72^

### Disorders of Amino Acid Metabolism

Four clinical subtypes of maple syrup urine disease (MSUD) are described. Classic MSUD presents soon after birth and is a severe and often rapidly lethal disorder. The phenotypes of the other subtypes (intermediate, intermittent, and thiamine‐responsive MSUD) are overlapping. Presentation in adulthood is very rare. Patients with MSUD may decompensate during catabolic states and develop behavioral changes, nausea, vomiting, and eventually coma attributed to cerebral edema. Movement disorders may also be present.^21^ Abnormalities of eye movements are described in infants and vary from up‐gaze palsy and adduction weakness to absence of voluntary eye movements with absent vestibulo‐ocular reflexes.^73^, ^74^, ^75^ Treatment is with a low‐protein diet in combination with a leucine‐, isoleucine‐, and valine‐free amino acid supplement. Emergency treatment is necessary during metabolic stress, such as intercurrent illnesses.^21^

Glutaric aciduria type 1 (GA1) usually begins in childhood, but adult‐onset has been reported as well. Catabolic episodes and intercurrent illnesses result in damage to the caudate nucleus and putamen, causing severe dystonia.^21^ Ocular abnormalities include intraretinal haemorrhages, cataract, and pigmentary retinopathy.^76^ A 19‐year‐old woman with GA1 showed horizontal nystagmus, upward gaze palsy, and paralysis of convergence.^77^ Other patients with gaze palsy have been described, but gaze palsy in these patients might be secondary to increased intracranial pressure attributed to the intracranial haemorrhages that may be present in GA1.^76^ Dietary treatment with a low‐lysine diet and carnitine supplementation prevents damage to the striatum. Similar to MSUD, emergency treatment is necessary to prevent catabolism during periods of fever or prolonged fasting.^21^

### Congenital Disorders of Glycosylation

Phosphomannomutase 2 deficiency (PMM2‐CDG or CDG1A) is the most common congenital disorder of glycosylation. The phenotype is variable, and multiple organs can be involved.^21^ PMM2‐CDG is usually diagnosed in childhood, but attenuated forms present later. In adulthood, the symptoms may be mild and include ataxia and learning difficulties.^78^ A whole range of ocular manifestations can occur and include strabismus, impaired smooth pursuit, nystagmus, ocular flutter, ocular motor apraxia, impaired optokinetic nystagmus, and impaired vestibulo‐ocular reflexes.^79^, ^80^, ^81^, ^82^ Strabismus and nystagmus might be secondary to visual impairment, although they are also described in patients with PMM2‐CDG who have normal vision.^78^, ^82^ Other subtypes of congenital disorders of glycosylation 1 are less common, but some of these patients also show strabismus and nystagmus.^82^ With the exception of a few subtypes of CDG syndromes, treatment is not available.

### Disorders of Purine or Pyrimidine Metabolism

Variants of Lesch Nyhan syndrome are described that present in early adulthood with symptoms of hyperuricemia, for example, nephrolithiasis, crystalluria, and gout.^21^ Ocular motor abnormalities are observed particularly in severe (early‐onset) HPRT deficiency and include impaired smooth pursuit and difficulty initiating voluntary saccades that appears as an ocular motor apraxia.^83^ Hyperuricemia must be treated.^21^

### Peroxisomal Disorders

In patients suffering from Zellweger spectrum disorders, three different presentations can be observed: a neonatal‐infantile, a childhood, and an adolescent‐adult presentation. The majority of patients presents in childhood.^84^ The phenotype is milder when the disease begins in adolescents or adults. Patients have mild‐to‐severe cognitive impairment in combination with retinal dystrophy, cataract, glaucoma, hearing impairment, ataxia, pyramidal symptoms, or peripheral neuropathy.^21^ Vision is frequently impaired.^84^ Ocular motor abnormalities include hypometric saccades (particularly in the horizontal plane), saccadic intrusions (square‐wave jerks), and gaze‐evoked nystagmus.^85^ Pendular nystagmus can be observed.^86^ No curative treatment is available.

### Neurotransmitter disorders

Disorders of neurotransmitters, especially those that affect the dopaminergic pathways, can cause dystonia with oculogyric crises. Response to low doses of l‐dopa in some of these diseases is excellent. Neurotransmitter disorders can be divided into those affecting synthesis, those affecting dopamine transport, and those affecting degradation.

Oculogyric crisis is frequently observed in disorders affecting dopamine synthesis, whereas other abnormalties of eye movements are rare in these disorders.^87^ Many of these disorders present early in life and, for most of the neurotransmitter disorders, late‐onset presentation is rare. Patients with milder forms of these disorders may remain undiagnosed until adolescence or adulthood, or may be mistakenly diagnosed with cerebral palsy.^21^ However, recognition of these disorders is important because patients can improve dramatically when treated properly. Most of the late‐onset neurotransmitter disorders are caused by autosomal dominant GTP‐CH‐I deficiency. Patients present with dystonia of the lower limbs that usually progresses to generalized dystonia, although the late‐onset form can also be associated with parkinsonism.^88^ Autosomal recessive forms of GTP‐CH‐I deficiency have also been described. Oculogyric crises are more frequent in recessive than dominant forms of GTP‐CH‐I deficiency.^89^, ^90^, ^91^ Patients with aromatic L‐amino acid decarboxylase deficiency (AADC),^21^, ^92^ tyrosine hydroxylase deficiency,^93^, ^94^, ^95^, ^96^ and 6‐pyruvoyl‐tetrahydropterin synthase deficiency^97^, ^98^ may show oculogyric crisis, in particular in AADC in which oculogyric crisis is one of the key features.^92^, ^99^, ^100^ Finally, oculogyric crisis is also described in sepiapterin reductase deficiency.^101^, ^102^, ^103^

Disorders affecting dopamine transport include brain dopamine‐serotonin vesicular disease (vesicular monoamine transporter 2 deficiency) and dopamine transporter deficiency syndrome (DAT deficiency). In the latter, adult onset is reported with parkinsonism and psychiatric symptoms.^104^ Both disorders are associated with oculogyric crisis.^87^, ^105^, ^106^ Other abnormalities of eye movements are also observed in DAT deficiency, including saccadic intrusions during smooth pursuit, saccadic oscillations (ocular flutter), slow saccadic eye movements, and ocular motor apraxia.^87^, ^104^, ^107^

### Energy Metabolism Disorders

Mitochondrial diseases are a group of disorders caused by mutations in mitochondrial DNA (mtDNA; either maternally inherited or de novo) or nuclear DNA (Mendelian inherited). Tissues with high energy needs are commonly affected, including brain, heart, and skeletal muscles. There is a wide range of clinical phenotypes, and onset varies widely. Neurological involvement causes movement disorders, psychomotor retardation or regression, epilepsy, muscle weakness, and migraine.^108^ Adult onset of mitochondrial disease is especially frequent in disorders caused by multiple deletions in mtDNA, probably attributed to accumulation of mtDNA defects.^108^ Ocular involvement is frequent, and both central and peripheral causes of eye movements can be present. A well‐known form of ocular motor dysfunction in mitochondrial disease is PEO, characterized by progressive bilateral ptosis and weakness of the extraocular muscles. PEO often occurs in association with other symptoms.^109^ When it is the sole feature, it is called chronic PEO. PEO is seen in Kearn‐Sayre's syndrome, Pearson's syndrome, and multiple disorders attributed to mitochondrial deletions or point mutations.^110^ In mitochondrial neurogastrointestinal encephalomyopathy, PEO is characterized by slow and hypometric saccades, particularly for saccades larger than 10 degrees, and abducting saccades are slower than adducting saccades.^111^ Central eye movement disorders are observed in patients with Leigh's syndrome (subacute necrotizing encephalomyelopathy). Patients with early‐onset disease show disorders similar to those attributed to thiamine deficieny, including gaze‐evoked nystagmus, impaired vestibular responses, internuclear ophthalmoplegia, and upbeat nystagmus switching to downbeat nystagmus during convergence.^9^, ^112^ A combination of PEO with central eye movement disorders has been described in POLG‐related disorders. The phenotype of POLG‐related disorders is variable; it ranges from severe and often lethal childhood forms to later‐onset forms with a continuum of overlapping phenotypes, including ophthalmoplegia.^113^ In addition to ophthalmoplegia, ptosis, gaze‐evoked nystagmus, rebound nystagmus, abnormalties of saccades (dysmetria and slowing), and impaired pursuit have been observed.^114^ Treatment of mitochondrial diseases is still limited and includes vitamins and cofactors.^21^

Pyruvate dehydrogenase deficiency is divided into different subtypes. An adult patient diagnosed with pyruvate dehydrogenase E1 deficiency showed parkinsonism, impaired up gaze, and jerky horizontal eye movements during pursuit.^115^ Pendular nystagmus, eye rolling, and ocular motor apraxia are reported in children with pyruvate dehydrogenase E2 deficiency.^116^ The disease can be treated with a ketogenic diet, thiamine, L‐carnitine, and α‐lipoic acid.^117^

## Conclusions

We have reviewed IEM in which the onset of symptoms can occur relatively late in life and in which ocular motor abnormalities can be a prominent sign. Recognition of these patterns of abnormalities of eye movements is important because they may be the key to accurate early diagnosis and thus to a timely start of treatment. Unfortunately, there continues to be a lack of information about eye movement disorders in many IEMs because little attention is given to them in daily practice. Examination of the vestibular system, in particular, is neglected in most studies even though it often provides essential information about localization and diagnosis. Disorders of hearing are commonly recognized in many IEMs, but vestibular function is rarely commented upon. A standard, focused examination of the different subtypes of eye movements (range of motion, gaze‐holding, saccades, pursuit, and vestibular responses) can be performed relatively quickly in most patients during routine physical examination. Testing with video‐oculography has also become more user‐ and patient‐friendly and helps to quantify the eye movement abnormalities, making these abnormalities a valuable biomarker for following the natural course of disease or the response to therapies.

## Author Roles

(1) Research Project: A. Conception, B. Organization, C. Execution; (2) Statistical Analysis: A. Design, B. Execution, C. Review and Critique; (3) Manuscript Preparation: A. Writing of the First Draft, B. Review and Critique.

L.H.K.: 1A, 1B, 1C, 3A, 3B

M.A.J.T.: 1A, 1B, 3B

F.L.: 3B

B.H.R.W.: 3B

A.R.: 1C, 3B

D.S.Z.: 1C, 3B

T.J.d.K.: 1A, 1B, 3B

## Financial Disclosures

M.A.J.T. reports grants from Dystonia Medical Research Foundation, grants from Stichting Wetenschapsfonds Dystonie Vereniging, grants from Prinses Beatrix Foundation, grants from Fonds NutsOhra, from Jacques and Gloria Gossweiler Foundation, grants from Fonds Psychische Gezondheid, grants from Phelps Stichting, and grants from Ipsen & Allergan Farmaceutics, Merz, Medtronic, and Actelion. D.S.Z. reports book royalties from Oxford University Press and honoraria for teaching in vestibular educational courses sponsored by Micromedical Technology. T.J.d.K. reports grants from Metabolic Power Foundation, grants from Friends of Beatrix Childrens Hospital, grants from Actelion pharmaceuticals, personal fees from Actelion pharmaceuticals, and personal fees from Nutricia Medical Nutrition.

## Supporting information

Supporting Information Appendix S1

Supporting Information Appendix S2

Video 1 (published before in Leigh)9:
This 25‐year‐old woman presented with an 8‐year history of progressive imbalance due to Niemann‐Pick type C disease. Horizontal saccades were relatively normal but, as shown in this video, vertical saccades were slow (especially downward) and showed curved trajectories.
