# Supplementary material for: Eye movement disorders and neurological symptoms in late‐onset inborn errors of metabolism
Source: Mov Disord. 2018 Nov 28;33(12):1844–56. doi: 10.1002/mds.27484 (PMC6587951; doi:10.1002/mds.27484)
Supplement: Supplementary file 2 — Supporting Information Appendix S2 [file MDS-33-1844-s002.docx]

**Supplementary appendix II: Reference list with videos of eye movement disorders in IEM**

**Niemann-Pick type C:**

“Round the houses” phenomenon:

Eggink Hendriekje H. Teaching Video NeuroImages: The "round the houses" sign as a clinical clue for Niemann-Pick disease type C. Neurology 2016;86:e202.

Impaired saccades and optokinetic nystagmus:

Leigh RJ, Zee DS. The neurology of eye movements. Ed. 5. New York: Oxford University Press; 2015.

**Glucose transporter type 1 deficiency:**

Paroxysmal eye movement abnormalities:

De Giorgis V, Varesio C, Baldassari C et al. Atypical Manifestations in Glut1 Deficiency Syndrome. J Child Neurol 2016;31:1174-1180.

Pearson TS, Pons R, Engelstad K, Kane SA, Goldberg ME, De Vivo DC. Paroxysmal eye-head movements in Glut1 deficiency syndrome. Neurology 2017; 88:1666-1673.

**Wilson’s disease:**

Selective slowing of downward saccades:

` Jung H, Choi SY, Kim J, Kim J. Selective slowing of downward saccades in Wilson's disease. Parkinsonism Relat Disord 2013;19:134-135.

**Pantothenate kinase-associated neurodegeneration:**

Supranuclear gaze palsy:

Bozi M, Matarin M, Theocharis I, Potagas C, Stefanis L. A patient with pantothenate kinase-associated neurodegeneration and supranuclear gaze palsy. Clin Neurol Neurosurg 2009;111:688-690.

**Adult-onset dystonia-parkinsonism:**

Ocylogyric crisis:

Virmani T, Thenganatt MA, Goldman JS, Kubisch C, Greene PE, Alcalay RN. Oculogyric crises induced by levodopa in PLA2G6 parkinsonism-dystonia. Parkinsonism Relat Disord 2014;20:245-247.

**Phosphomannomutase 2 deficiency:**

Impaired smooth pursuit, nystagmus, and impaired optokinetic nystagmus:

Coorg R, Lotze TE. Child Neurology: a case of PMM2-CDG (CDG 1a) presenting with unusual eye movements. Neurology 2012;79:131.

**Neurotransmitter disorders:**

Ocular flutter and impaired saccades:

Kurian MA, Li Y, Zhen J et al. Clinical and molecular characterisation of hereditary dopamine transporter deficiency syndrome: an observational cohort and experimental study. Lancet Neurol 2011;10:54-62.

Saccades with increased latency in initiation, suppressible head movements to initiate, and moderate slowing of movement velocity:

Hansen FH, Skjørringe T, Yasmeen S et al. Missense dopamine transporter mutations associate with adult parkinsonism and ADHD. J Clin Invest 2014;124:3107-3120.

**Mitochondrial diseases:**

Chronic progressive external ophthalmoplegia:

Leigh RJ, Zee DS. The neurology of eye movements. Ed. 5. New York: Oxford University Press; 2015
